# The Effect of Text Message-Based mHealth Interventions on Physical Activity and Weight Loss: A Systematic Review and Meta-Analysis

**DOI:** 10.1177/15598276241268324

**Published:** 2024-08-15

**Authors:** Aminah Emeran, Robyn Burrows, Josh Loyson, Muhammed Rizaan Behardien, Lauren Wiemers, Estelle Lambert

**Affiliations:** 1 UCT Research Centre for Health Through Physical Activity Lifestyle and Sport (HPALS), Department of Human Biology, Faculty of Health Sciences, 37716 University of Cape Town, Cape Town, South Africa (AE, EL, RB, MRB, LW); 2 International Federation of Sports Medicine (FIMS) Collaborative Centre of Sports Medicine, 37716 University of Cape Town, Cape Town, South Africa (AE, EL, RB, MRB); 3 Department of Radiation Medicine, Division of Radiobiology, Faculty of Health Sciences, 37716 University of Cape Town, Cape Town, South Africa (JL)

**Keywords:** mobile health, e-Health, non-communicable diseases, behavior change

## Abstract

Physical inactivity and obesity are detrimental to one’s overall health, as they increase the risk of developing non-communicable diseases. Fortunately, physical inactivity and obesity can be improved by supporting lifestyle behavior changes. This support may be provided remotely by Mobile Health (mHealth) messaging interventions, which involve using mobile messages for health improvement. This study aimed to determine the effect of mHealth interventions using unidirectional text messaging/instant messaging on physical activity and weight-related outcomes in adult populations. An electronic literature search was conducted using PubMed, Scopus, and Web of Science, for pre-post interventions using unidirectional messaging for physical activity/weight loss. A total of 43 articles were included in the review. Most studies used non-tailored text messages, were RCTs, and were performed on clinical populations in high income countries. Meta-analysis showed that messages had minimal effects on physical activity (d+: .14, 95% CI: .05 to .23, *P* = .003, I^2^ = 65%), and no effect on weight loss (d+: .04, 95% CI: −.02 to .10, *P* = .21, I^2^ = 29%). This suggests that unidirectional messaging alone is not sufficient to promote physical activity and weight loss. Future studies should consider using bidirectional messaging or other interventions in addition to messages, such as mobile applications, to potentially improve intervention effectiveness.

“Our review included unidirectional, text message-only interventions, and found limited effects on physical activity and weight loss outcomes.”

## Introduction

Physical inactivity and obesity remain significant public health concerns globally. Approximately 28% of the global population do not meet minimum physical activity requirements,^1,2^ while 38% of people worldwide are overweight or obese.^3^ Physical inactivity and obesity are detrimental to one’s overall health, as they may increase the risk of developing non-communicable diseases (NCDs) such as diabetes and cardiovascular disease.^4,5^ NCDs place a substantial burden on global mortality, with more than 74% of deaths worldwide being accounted for by NCDs.^4^

Fortunately, physical inactivity and obesity are modifiable risk factors of NCDs, and thus can be improved through public health interventions promoting healthy lifestyle changes.^4^ An example of a promising NCD intervention medium, which use has increased substantially within the past few years, is mobile health or mHealth interventions.^1^

MHealth refers to the use of mobile technologies such as mobile applications and text messaging, to promote or provide health care at a distance.^6^ It is appealing to many due to its potentially low cost, accessibility, and scalability.^7^

Several systematic reviews and meta-analyses have reported positive effects of mHealth interventions on NCD risk factors, including physical activity and weight management.^7-10^ A systematic review by Pfaeffli Dale et al, found an overall positive effect of mHealth interventions on increasing physical activity behavior.^11^ While a systematic review and meta-analysis by Buss and colleagues, observed weight loss and decreased BMI as their most successfully reported results.^10^

Despite some positive findings, many reviews highlight that the evidence of mHealth use for health behavior change is limited. This is due to small effect sizes and low-quality studies.^8,10^ Additionally, heterogeneity of studies is high due to a wide range possible mHealth mediums and health outcome measurements used.^11-13^

Although there is a wide range of possible mHealth modalities, text messaging is one of the most frequently used mHealth mediums.^6^ Systematic reviews on text message-based mHealth interventions report mostly positive, small effect sizes on health outcomes including physical activity.^6,14^ However, with the increased use of instant messaging services such as WhatsApp and WeChat, SMS use has declined.^15^ Instant messaging differs from text messaging, as it relies on Internet connection as opposed to mobile network connectivity. Furthermore, instant messaging has a broader range of functionalities, including the ability to share much larger messages and files, and enabling the exchange of various media formats, such as images, videos, documents, and voice messages.^16^ However, both instant messaging and text messaging enable two-way communication between users.

Although both instant messaging and text messaging allow for bidirectional communication, one-way messaging is potentially a simpler and more cost-effective method to implement messaging into mHealth interventions, as it does not require participants to respond to intervention messages.^17,18^ Unidirectional messaging has also shown effectiveness in promoting weight loss and has been used in several previous mHealth studies.^18-20^

Other characteristics that may differ between mobile message interventions include differences in message content, message tailoring, and use of theory to develop the messages. These factors are important to consider, as they may impact the effectiveness of messaging interventions.^14^

Considering the high prevalence of physical inactivity and obesity, the potential benefits of unidirectional messaging interventions, and the increased use of instant messaging, we conducted a systematic review and meta-analysis to determine the effectiveness of unidirectional text messaging and instant messaging interventions on physical activity and weight management. Secondly, we aimed to explore factors that may influence the effectiveness of the interventions, such as the type of messages (theory-based, tailored), frequency of messages and study duration.

As far as we are aware, no systematic review and meta-analysis has been conducted on studies using unidirectional SMS and/or instant messaging and measuring physical activity and weight loss as health outcomes. Thus, this review and meta-analysis will add to the existing body of evidence on the effectiveness of mHealth interventions on physical activity and weight loss.

## Methods

### Overview

This systematic review was conducted following the Preferred Reporting Items for Systematic Reviews and Meta-Analyses (PRISMA) guidelines.

The review protocol was registered in The International Platform of Registered Systematic Review and Meta-analysis Protocols (registration number INPLASY202370072). The review protocol can be accessed via the INPLASY website.

### Search Strategy

An electronic literature search was conducted between November 2022 and November 2023 searching the databases PubMed, Scopus, and Web of Science. We searched for literature on mHealth text messaging and/or instant messaging interventions targeting physical activity and/or weight loss. Search terms included: mHealth, eHealth, physical activity, exercise, weight loss, interventions, and programs. The full search strategy with Boolean operators can be viewed in Appendix A.

Studies were included if they were published between the years 2010 and 2023. This date range was used as we believed that the most relevant articles for our review would be found within the past few years, due to the rapid growth of mHealth and mobile technology development in recent years.^13,21^ Only articles available in English were included due to English speaking reviewers. Non-clinical and clinical populations with NCDs were included. Pregnant women were also included.

### Eligibility Criteria

Eligible study designs were pre-post intervention studies (randomized control trials (RCTs), non-RCTs, quasi experimental and mixed method studies) with a comparator group that received either no intervention, or “standard-of-care” treatment. Control groups that received neutral, generic messages were also included as this was considered “standard of care.”

Eligible interventions included mHealth interventions that used text messaging and/or instant messaging to send one-way motivational, educational or advice messages to participants to improve physical activity levels and/or body weight. Message content was classified according to the respective authors classification, as well as additional criteria. Motivational content included messages with encouraging, positively reinforcing language. Educational content included informative content on a particular health topic, while advice messages had suggested tips to improve lifestyle behaviors.

Studies using messages only as prompts, reminders, or health feedback were excluded. However, if these messages were in combination with motivational or educational messages, they were included in the review. If the study had other intervention components in addition to the messages or used two-way messages, they were excluded. Interventions of any duration were included. Interventions that sent messages via an instant messaging application group chat as opposed to individually sent messages were also excluded. The above criteria were chosen to reduce the heterogeneity of the included studies.

### Outcomes

Outcomes were grouped into (a) physical activity-related outcomes and (b) weight loss-related outcomes. Because the studies had a range of measures for physical activity and weight loss, specific measures were included in the following order of hierarchy, based on frequency of use:

(a) physical activity: (i) metabolic equivalent of tasks (METs), (ii) moderate/vigorous physical activity (MVPA), (iii) step count, (iv) minutes of exercise per week. When studies only had one measure for physical activity, that measure was used. These included sedentary time, number of sessions per week, adherence to guidelines, and physical activity score.
(b) weight loss-related outcomes: (i) body mass index (BMI), (ii) change in body weight in kg, (iii) percentage weight change. In one study, adherence to weight guidelines was used as this was the only measure of weight performed.^22^

Outcomes were eligible if they were self-reported or objectively measured, and if they were primary or secondary outcomes. Studies were included if they had only one or a combination of the above outcomes.

### Data Collection Process

Article titles and abstracts of all papers found during the literature search were downloaded and exported into *Rayyan Intelligent Systematic Review* website (https://www.rayyan.ai/)^23^ for screening. One reviewer screened the titles for eligibility (AE). Thereafter, 2 reviewers independently screened the abstracts for eligibility (AE and VL/LW/JL/MRB). In some cases, the abstract did not provide enough information to make an inclusion decision, and reviewers would screen the full text for eligibility. Once all reviewers had completed the screening, conflicting decisions were assessed and discussed, and a group decision was made whether to include or exclude the study. After abstract screening, 2 reviewers screened the articles for full text eligibility (AE and JL).

### Data Extraction and Management

Data was extracted independently by 2 investigators (AE and RB) according to a study specific excel spreadsheet. Conflicts were reviewed and resolved between the 2 investigators. The following information were included in the data extraction table: (i) primary author, (ii) study characteristics such as study design and duration, (iii) population characteristics such as age, sex, and country economic status, (iv) intervention characteristics such as outcomes measured, comparator, mode of message, message content, whether the messages were tailored or non-tailored, message frequency, whether messages were linked to theory, and (iv) effect sizes (standardized mean differences) of each outcome. Tailoring was defined as “any combination of strategies and information intended to reach one specific person, based on characteristics that are unique to that person, related to the outcome of interest, and derived from an individual assessment.”^24^ The economic status of countries was determined using The World Bank data.^25^

When studies had both self-reported and objective outcome data, the objective data were extracted. Additionally, when studies had more than one intervention group, only one intervention group was included. For example, one study had 2 intervention groups receiving either promotion or prevention-based messages. In this case, the first intervention group mentioned in the study (promotion messages) was included in the review.^26^ Another study had 2 intervention groups with different study durations. The group with the longer duration (20 weeks) was chosen.^27^ This was done to avoid “double counting” of participants in the control groups, as both intervention groups were being compared to the same control group.^28^

## Statistical Analysis

IBM SPSS version 28.0.1.1 was used to summarize study descriptive statistics and for analysis of potential effect moderators. Statistical tests used in SPSS included the Pearson Chi squared test, independent *t* test, and Pearson correlation coefficient test.

Meta-Analyses were conducted using JASP Software Version 0.17.21 (University of Amsterdam). A Random effects model was used to calculate weighted averages and pooled estimates due to likely variance in included studies.

Standardized mean difference (SMD) as Hedges g was used as a measure of effect size. SMD was calculated for continuous data by extracting each study’s intervention and control group sample sizes, and pre-post intervention mean changes with the standard deviations (SD) into an excel spreadsheet. When studies used standard error or 95% confidence intervals instead of SD, these values were converted into SD. If the study only used median values, the study was omitted from meta-analysis. Negative values for weight change were adjusted into positive values to ensure improvement of outcomes were in the same direction. When studies had multiple time points for the outcome measurement, the final time point at the end of the intervention was used for SMD calculation.

Once effect sizes for each study were calculated, it was uploaded into JASP software for meta-analysis. A 95% Confidence Interval was used as an estimate of effect size with statistical significance set at a *P*-value of <.05. Heterogeneity was measured using the I^2^ statistic. A Funnel plot analysis was used to measure publication and reporting bias.

Two sensitivity analyses were conducted to determine the robustness of the primary results, by removing outlier studies and non-RCT studies.

The overall meta-analysis results were evaluated and scored using the Grading of Recommendations Assessment, Development, and Evaluation (GRADE).^29^

### Studies Critical Appraisal

The Mixed Method Appraisal Tool (MMAT) version 2018 was used to assess the methodological quality of selected studies.^30^ This tool was selected as it allows for the critical appraisal of a combination of different study designs including qualitative randomized controlled trials, quantitative non-randomized trials, mixed method studies, and qualitative studies. MMAT includes 2 general screening questions before further appraisal can be performed, and 5 questions specific to each study design, which must be answered with “Yes,” “No” or “Can’t tell” if not enough information is present. The tool discourages generating an overall quality score for each study, but rather provides a detailed analysis of each criterion. MMAT was applied to each study by 2 independent authors (AE and RB) for quality appraisal. When conflicts were present, they were highlighted and resolved between authors.

## Results

**Figure 1.**
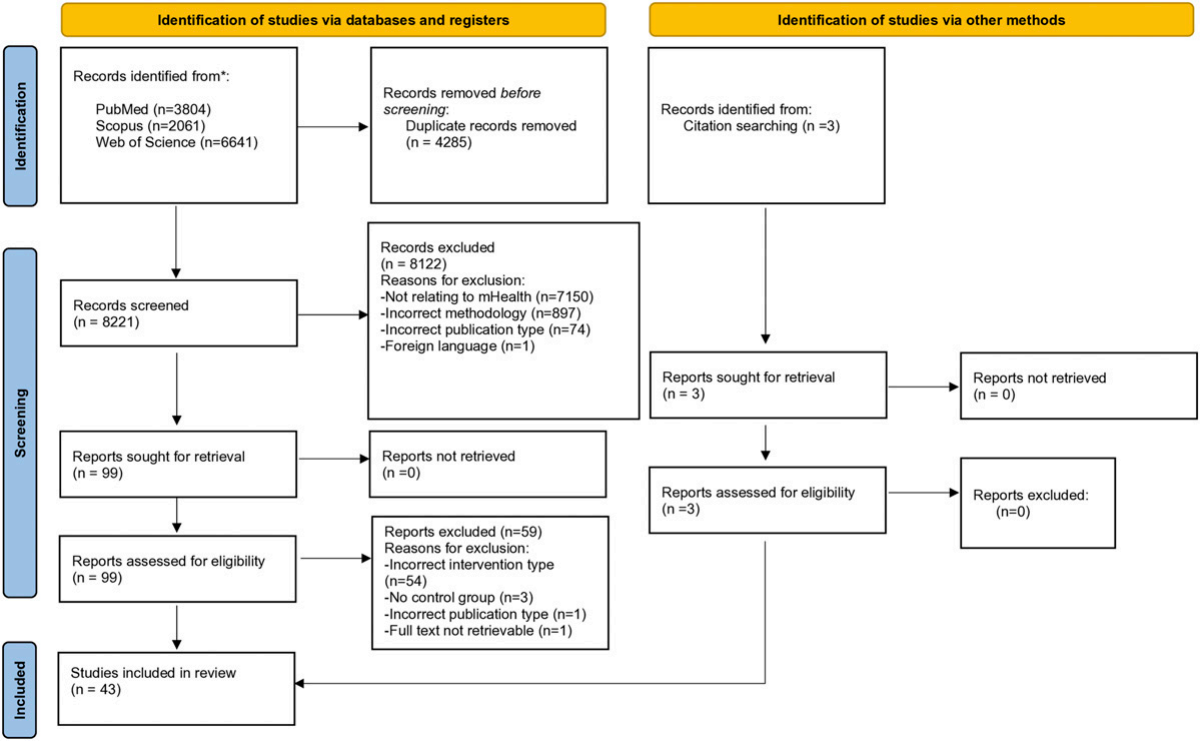
Flow diagram of study selection.

A total of 8221 records were identified across the 3 journals in the database search, following removal of 4285 duplicates. After screening of titles, 991 publications were identified for abstract screening. Most of the excluded articles were unrelated to mHealth and behavior change. Abstract screening identified 99 articles, with articles being excluded due to not meeting the inclusion criteria. After reviewing full texts for eligibility, 40 records were chosen for inclusion. Several studies that initially appeared to meet all the inclusion criteria, were excluding after full text review, due to having bidirectional messaging.^31,32^ Three additional articles were identified during full text reference screening, leaving 43 articles meeting all the inclusion criteria for systematic review. Study screening and selection is summarized in the PRISMA flow diagram (Figure 1).

### Characteristics of Included Studies

Study characteristics are summarized in Table 1. Studies were published between the years 2013 to 2023. Most studies were performed on a clinical population (n = 31), with the most common population condition being persons living with diabetes and obesity.

**Table 1.** Characteristics of Included Studies (N = 43).

First Author	Study Design	Sample Size	Study Duration (months)	Participant Characteristics	Outcomes of Interest	Tailored/Non-Tailored Messages	Intervention Linked to Theory	Frequency of Messages	Results
Kim and Glanz^33^	RCT	IG: 26 C: 10 Per protocol	1.5	*Age*: 70 *Percent Male*: 20 *Population condition*: Diabetes and hypertension *Country type*: HIC	Steps per day	Non-tailored	None	>7 per week	Improvement in steps per day in intervention compared to control. (IG: +679 steps vs C: +398 steps, *P* < .05)
Tamban et al^22^	RCT	IG: 46 C: 36 ITT	6	*Age*: 50 *Percent Male*: 27 *Population condition*: Diabetes *Country type*: LMIC	Adherence to PA guidelines and BMI	Non-tailored	None	2-4 per week	Improvement in adherence to exercise in minutes (IG: 37.40 + 14.87, C: 31.44 + 10.82) (*P* = .02)
Shaw et al^26^	RCT	IG: 41 C: 39 ITT	3	*Age*: 53 *Percent male*: 74 *Population condition*: Overweight/obesity *Country type*: HIC	Weight change	Non-tailored	Regulatory focus theory	Daily	No improvement in weight (*P* = .22)
Muñoz et al^34^	RCT	IG: 59 C: 55 Per protocol	4	*Age*: 21 *Percent male*: 36 *Population condition*: Non-clinical *Country type*: HIC	Steps per day and BMI	Non-tailored	None	2-4 per week	The intervention and control groups did not differ significantly in BMI, and physical activity (*P* = .467)
Ahn and Choi^19^	RCT	IG: 25 C: 29 Per protocol	3	*Age*: 49 *Percent male*: 50 *Population condition*: Overweight/obesity *Country type*: HIC	BMI	Non-tailored	None	2-4 per week	Improvement in BMI (IG: 27.9 vs C: 28.3, *P* = .02)
Chow et al^35^	RCT	IG: 352 C: 358 ITT	6	*Age*: 58 *Percent Male*: 82 *Population condition*: CVD Country type: HIC	METS and BMI	Tailored	None	2-4 per week	Decrease in BMI and increase in METS in intervention group compared to the control group (*P* < .001)
Kim et al^36^	RCT	IG: 101 C: 95	6	*Age*: 41 *Percent Male*: 100 *Population condition*: Overweight/obesity *Country type*: HIC	METS and weight change (kg)	Tailored	None	2-4 per week	Decrease in weight in intervention (−1.71 kg) and control group (−1.56 kg). However, no difference between groups (*P* = .78). Increase in METS in intervention group (*P* < .008). However, no between group difference (*P* = .14)
Gell and Wadsworth^37^	RCT	IG: 37 C: 37 ITT	6	*Age*: 47 *Percent male*: 0 *Population condition*: Non-clinical *Country type*: HIC	Steps per day	Non-tailored	None	2-4 per week	Difference in steps between groups at 12 weeks (6540.0 vs 5685.0, *P* = .01). No statistically significant difference in steps per day between groups at 24 weeks (6867.7 vs 6189.0, *P* = .06)
Martin et al^38^	RCT	IG: 16 C: 16 ITT	0.5	*Age*: 58 *Percent male*: 54 *Population condition*: CVD *Country type*: HIC	Steps per day	Tailored	Behaviour change theory	2-4 per week	Statistically significant improvement in steps per day between groups. Mean difference between groups: 2534 (1318-3750), *P* < .001
Cotten and Prapavessis ^39^	RCT	IG: 41 C: 41 ITT	1.5	*Age*: 21 *Percent male*: 26 *Population condition*: Non-clinical *Country type*: HIC	Moderate physical activity	Non-tailored	None	>7 per week	Increase in moderate PA in intervention group (*P* = .02) However, no difference in PA between groups. (*P* = .16)
Kinnafick et al^40^	RCT	IG: 34 C: 31 ITT	2.5	*Age*: 25 *Population Percent male*: 6 *Population condition*: Non-clinical *Country type*: HIC	Moderate physical activity	Tailored	Self-determination theory	2-4 per week	No improvement in MPA (*P* = .06)
Peimani et al^41^	RCT	IG: 50 C: 50 Per protocol	3	*Age*: 53 *Percent Male*: 54 *Population condition*: Diabetes *Country type*: LMIC	BMI	Tailored	Social cognitive theory	Daily	Statistically significant decrease in BMI in the intervention group compared to control group (*P* < .001)
Abaza and Marschollek^42^	RCT	IG: 34 C: 39 Per protocol	3	*Age*: 52 *Percent Male*: 44 *Population condition*: Diabetes *Country type*: LMIC	Weight change (kg)	Non-tailored	None	Daily	Intervention group had a decrease in weight of 1.3 kg and control group decreased by 0.5 kg. However, no statistically significant difference in weight between groups (*P* = .22)
McCoy et al^43^	Quasi experimental	IG: 50 C: 27 Per protocol	2.5	*Age*: 53 *Percent Male*: 13 *Population condition*: Overweight/Obesity *Country type*: HIC	Minutes of exercise per day	Non-tailored	Health belief and IMB model	2-4 per week	Intervention participants increased exercise time by 8% (*P* = .03), while the control group’s exercise time remained constant. However, no statistically significant difference between groups
Fang and Deng^44^	RCT	IG: 58 C: 51 Per protocol	12	*Age*: 57 *Percent Male*: 86 *Population condition*: Diabetes *Country type*: UMIC	BMI	Non-tailored	None	1 per month	No statistically significant difference in BMI between groups (*P* = .80)
Silina et al^45^	RCT	IG: 63 C: 60 Per protocol	12	*Age*: 37 *Percent male*: 29 *Population condition*: Overweight/obesity *Country type*: HIC	BMI	Non-tailored	Ideas of planned behaviour and social cognitive theory	1 every 2 weeks	Statistically significant improvement in BMI (mean difference −1.14 (95% CI –1.9, −.41), *P* = .002)
Lemanu et al^46^	RCT	IG: 44 C: 44 Per protocol	1.5	*Age*: 44 *Percent Male*: 69 *Population condition*: Overweight/obesity *Country type*: HIC	METS	Non-tailored	None	Daily	Intervention had increase in METS. However, no difference between groups (*P* = .27)
Abbaspoor et al^47^	RCT	IG: 44 C: 44 Per protocol	3	*Age*: 29 *Percent male*: 0 *Population condition*: Pregnancy and prediabetes *Country type*: LMIC	METS	Non-tailored	None	2-4 per week	Improvement in METS (IG: 852.42 ± 350 vs C: 637.12±210, *P* < .001)
Boels et al^48^	RCT	IG: 114 C: 115 ITT	6	*Age*: 59 *Percent Male*: 60 *Population condition*: Diabetes *Country type*: HIC	METS and BMI	Tailored	Fogg behaviour model	Varied	No improvements in BMI (*P* = .95) or METS (*P* = .65)
Owolabi et al^49^	RCT	IG: 108 C: 108 ITT	6	*Age*: 60 *Percent Male*: 17 *Population condition*: Diabetes *Country type*: UMIC	BMI	Non-tailored	Behaviour change theory	Daily	No statistically significant difference in BMI between groups (*P* = .44)
Nepper et al^50^	Quasi experimental	IG: 35 C: 35 Per protocol	3	*Age*: 57 *Percent Male*: 34 *Population condition*: Diabetes *Country type*: HIC	METS	Non-tailored	None	2-4 per week	Statistically significant increase in METS for intervention group compared to control group (*P* = .02)
Gore et al^51^	Quasi experimental	IG: 204 C: 208 Per protocol	12	*Age*: 50 *Percent male*: 40 *Population condition*: Non-clinical *Country type*: HIC	BMI	Non-tailored	None	Daily	No improvement in BMI (*P* = .92)
Vinitha et al^52^	RCT	IG: 126 C: 122 ITT	24	*Age*: 43 *Percent Male*: 68 *Population condition*: Diabetes *Country type*: LMIC	BMI	Non-tailored	None	2-4 per week	No improvement in BMI (*P* = .58)
Chan et al^53^	RCT	IG: 18 C: 15 Per protocol	2	*Age*: 38 *Percent male*: 16 *Population condition*: Non-clinical *Country type*: HIC	BMI	Non-tailored	None	2-4 per week	No improvements in BMI (*P* = .22)
Keating and McCurry^54^	Mixed methods	IG: 50 C: 45 Per protocol	2	*Age*: 21 *Percent male*: 21 *Population condition*: Overweight/obesity *Country type*: HIC	BMI	Non-tailored	Transtheoretical model	Daily	No improvement in BMI (p-.83)
Huo et al^55^	RCT	IG:251 C:251 ITT	6	*Age*: 60 *Percent Male*: 83 *Population condition*: CVD and diabetes *Country type*: UMIC	METS and BMI	Non-tailored	Behaviour change theory	5-6 per week	No statistically significant difference in METS (*P* = .78) and BMI (*P* = .21) between groups
Nanditha et al^56^	RCT	IG:1031 C:1031 ITT	24	*Age*: 52 Percent male: 64 *Population condition*: Prediabetes *Country type*: HIC and LMIC	MVPA and BMI	Tailored	Transtheoretical and health belief model	2-4 per week	No statistically significant difference in physical activity and BMI between groups
Bolmsjö et al^57^	RCT	IG:29 C:28 Per protocol	6	*Age*: 67 *Percent Male*: 44 *Population condition*: Hypertension *Country type*: HIC	BMI	Non-tailored	None	2-4 per week	No improvement in BMI (*P* = .13)
Holmes et al^58^	RCT	IG:38 C: 34 Per protocol	4.5	*Age*: 27 *Percent male*: 0 *Population condition*: Pregnancy *Country type*: HIC	Gestational weight guidelines	Non-tailored	Social cognitive theory	1 per week	No improvement in weight (*P* = .37)
Sadanshiv et al^59^	RCT	IG:161 C:159 ITT	3	*Age*: 48 *Percent Male*: 55 *Population condition*: Diabetes *Country type*: LMIC	BMI	Non-tailored	Transtheoretical model	2-4 per week	Decrease in BMI in IG compared to control. (Mean difference: −0.6, *P* < .001)
Ritchie et al^60^	Non-RCT	IG:236 C:252 ITT	12	*Age*: 50 *Percent Male*: 26 *Population condition*: Prediabetes *Country type*: HIC	Percent weight less	Non-tailored	None	5-6 per week	No improvement in weight (*P* = .37)
Farmer et al^61^	RCT	IG:510 C:511 Per protocol	12	*Age*: 57 *Percent Male*: 30 *Population condition*: Diabetes *Country type*: UMIC	BMI	Non-tailored	Behaviour change wheel	2-4 per week	No improvement in BMI (*P* = .67)
Waller et al^18^	RCT	IG:186 C:189 Per protocol	6	*Age*: 62 *Percent Male*: 51 *Population condition*: Diabetes *Country type*: HIC	PA sessions per week and BMI	Non-tailored	Behaviour change wheel	Varied	No improvement in PA (*P* = 0.19) or BMI (*P* = 0.51)
Saldivar et al^27^	Non-RCT	IG:94 C186 Per protocol	5	*Age*: 54 *Percent Male*: 17 *Population condition*: Overweight/obesity *Country type*: HIC	Weight change (kg)	Tailored	None	2-4 per week	Both groups had reduction in weight (C: 114 ± 27 kg vs 112 ± 26 kg, *P* < 0.001; IG: 111 ± 28 kg vs 109 ± 28 kg, *P* < .01) No difference between groups (*P* = .25)
Bootwong and Intarut^62^	RCT	IG:160 C:162 Per protocol	3	*Age*: 40 to over 50 *Percent Male*: 44 *Population condition*: Prediabetes *Country type*: UMIC	METS and BMI	Non-tailored	None	5-6 per week	Improvement in MET at 6 weeks (*P* = .02), but not at 12 weeks (*P* = .51) No difference in BMI (*P* = .36)
Singleton et al^63^	RCT	IG:78 C:78 ITT	6	*Age*: 56 *Percent Male*: 0 *Population condition*: Cancer *Country type*: HIC	METS and BMI	Non-tailored	None	2-4 per week	No statistically significant difference in METS (*P* = .79) and BMI between groups
Franco et al^64^	RCT	IG:96 C:91 Per protocol	3	*Age*: 43 *Percent Male*: 11 *Population condition*: Non-clinical *Country type*: HIC	PA score and BMI	Tailored	None	2-4 per week	No statistically significant difference in PA (*P* = .70) and BMI (*P* = .79) between groups
Zamanillo-Campos et al^65^	RCT	IG:89 C:90 ITT	3	*Age*: 62 *Percent Male*: 65 *Population condition*: Diabetes *Country type*: HIC	PA adherence	Tailored	Yes, type not specified	5-6 per week	No difference in physical activity levels (*P* = .10)
Kellner et al^66^	RCT	IG:16 C:18 Per protocol	0.5	*Age*: 22 *Percent Male*: 15 *Population condition*: Non-clinical *Country type*: HIC	Sedentary time	Non-tailored	Theory of action- and coping planning	Daily	Reduction in sedentary time in intervention group (*P* = .03), with no group effect (*P* = .12)
Kellner and Faas^67^	RCT	IG:41 C:31 Per protocol	1.5	*Age*: 21-24 *Percent Male*: 11% *Population condition*: Non-clinical *Country type*: HIC	Sedentary time	Non-tailored	Theory of action- and coping planning	Daily	Statistically significant decrease in sedentary time in intervention group compared to control (*P* = .43)
Golbus et al^68^	RCT	IG:97 C:97 ITT	6	*Age*: 60 *Percent Male*: 69 *Population condition*: CVD *Country type*: HIC	Steps per day	Tailored	None	Daily	No difference in steps in intervention compared to control group at 6 months (*P* = .32)
Acevedo et al^20^	RCT	IG:278 C:242 ITT	9	*Age*: 52 *Percent Male*: 0 *Population Condition*: CVD *County type*: HIC	Adherence to ideal cardiovascular BMI and PA guidelines	Non-tailored	Social cognitive theory	1 per week	No difference between intervention and control groups in BMI (*P* = .38) and PA (*P* = 1)
Huang et al^69^.	Quasi experimental	IG:89 C:105	2	*Age*: *39 (on next line-Percent male:50)* *Population condition*: none *Country type*: HIC	METS	Tailored	Theory of planned behaviour	1 per week	Statistically significant increase in METS in the intervention group compared to the control group (*P* = .02)

RCT, Randomised controlled Trial; IG, intervention group; C, Control; LMIC, lower middle-income country; UMIC, upper middle-income country; HIC, High income country; METS, Metabolic equivalent of tasks; MVPA, moderate to vigorous physical activity; BMI, body mass index; PA, physical activity; IMB model, The Information–Motivation–Behavioral Skills Model; CVD, cardiovascular disease; ITT, intention to treat.

Age presented as mean or median; results considered improved if statistically significant change in outcome values between groups; *P* < .05).

Mean age of participants was 47 years, with 37% of participants being male. Most studies were RCTs (n = 36), with the remaining being non-randomized (n = 2), quasi experimental (n = 4), and mixed method studies (n = 1). Study durations ranged between 2 weeks and 24 months, with the most frequent length being 6 months (n = 10). Sample sizes ranged from 10 participants to 1031 participants, with a median of 55 participants for the intervention group and 50 for the control group.

Physical activity was measured in 26 studies, was self-reported in 20 studies, and was most frequently reported in METS min/week (n = 10). The remaining studies used steps per day (n = 5), moderate to vigorous activity (n = 3), minutes of exercise per day (n = 1), sedentary time (n = 2), adherence to guidelines (n = 2), physical activity sessions per week (n = 1), and physical activity score (n = 1).

A total of 29 studies measured weight-related outcomes, with outcomes being objectively measured in 24 of the included studies. Outcomes were reported as BMI in 23 studies, weight change in 4 studies, percent weight loss in one study, and adherence to weight guidelines in one study.

Most studies used text messages/SMS as the mode of message delivery. The remaining used different forms of instant messaging to deliver messages, including smart phone application push messages (n = 1), messenger-based applications (n = 2), and web-based applications (n = 1).

Most of the studies used messages that were non-tailored (n = 31) and were not based on theory (n = 23). Messages were sent most frequently 2-4 times per week (n = 20), with the lowest number of messages being one per month, and the most messages being more than 7 per week. Message content most frequently included a combination of educational, advice-based, and motivational content on physical activity and healthy eating, followed by general disease information (e.g., diabetes and cardiovascular risk factors).

#### Effect of Interventions on Physical Activity and Weight-Related Outcomes

Only 8 of the 26 studies observed statistically significant improvements in physical activity in the intervention group compared to the control group, with a mean effect size of .23 (SD ± .38). Two of these studies showed improvements in objectively measured steps per day,^33,38^ three in self-reported METS min/week,^35,47,50^ and one in self-reported minutes of exercise per day.^22^ Several studies had statistically significant increases in physical activity of the intervention group, without a between-group effect.^36,39,43,46^

For weight-related outcomes, 5 out of 29 studies showed statistically significant improvements in BMI between groups, with a mean effect size of .07 (SD ± .14).^19,35,41,45,59^ Three studies had improvements in weight-related outcomes in intervention groups, with no statistically significant difference between groups.^27,36,42^

Analyses were conducted on potential effect moderators including message tailoring, primary vs secondary outcomes, theory-based messages, objective vs self-reported outcomes, message frequency and study duration.

The mean effect sizes of physical activity were slightly higher when messages were tailored (tailored: .29 vs non-tailored: .16), when outcomes were primary (primary: .32 vs secondary .09), when messages were based on theory (theory: .28 vs no theory: .18), and when outcomes were objectively measured vs self-reported (.41 vs .14). However, these differences were not statistically significant.

There were no statistically significant differences between effect size and message frequency, and no correlation between effect size and study duration. No differences in effect moderators for weight-related outcomes were found.

## Quality Appraisal

Table 2 shows a summary of the MMAT appraisal results of each study.

**Table 2.** MMAT Appraisal Tool.

Study	Randomized Quantitative (n = 36)							MMAT Scoring
	S1	S2	Q1	Q2	Q3	Q4	Q5	
Kim and Glanz^33^	Y	Y	N	Y	N	N	N	3/7
Tamban et al^22^	Y	Y	Y	Y	Y	Y	Y	7/7
Shaw et al^26^	Y	Y	C	Y	Y	C	Y	5/7
Muñoz et al^34^	Y	Y	C	Y	N	C	N	3/7
Ahn and Choi^19^	Y	Y	Y	Y	N	C	N	4/7
Chow et al^35^	Y	Y	Y	Y	Y	Y	Y	7/7
Kim et al^36^	Y	Y	C	Y	N	N	N	3/7
Gell and Wadsworth^37^	Y	Y	C	Y	Y	C	Y	5/7
Martin et al^38^	Y	Y	C	Y	Y	C	Y	5/7
Cotten and Prapavessis^39^	Y	Y	C	Y	N	C	N	3/7
Kinnafick et al^40^	Y	Y	C	Y	Y	Y	Y	6/7
Peimani et al^41^	Y	Y	C	Y	Y	N	Y	5/7
Abaza and Marschollek^42^	Y	Y	C	Y	Y	Y	Y	6/7
Fang and Deng^44^	Y	Y	C	Y	Y	C	Y	5/7
Silina et al^45^	Y	Y	C	Y	Y	C	Y	5/7
Lemanu et al^46^	Y	Y	Y	Y	Y	Y	Y	7/7
Abbaspoor et al^47^	Y	Y	C	Y	Y	C	Y	5/7
Boels et al^48^	Y	Y	C	Y	Y	N	Y	5/7
Owolabi et al^49^	Y	Y	C	Y	N	Y	N	4/7
Vinitha et al^52^	Y	Y	C	Y	Y	Y	Y	6/7
Chan et al^53^	Y	Y	N	N	N	N	N	2/7
Huo et al^55^	Y	Y	Y	Y	Y	Y	Y	7/7
Nanditha et al^56^	Y	Y	C	Y	Y	Y	Y	6/7
Bolmsjö et al^57^	Y	Y	Y	Y	Y	Y	Y	7/7
Holmes et al^58^	Y	Y	Y	Y	Y	N	Y	6/7
Sadanshiv et al^59^	Y	Y	Y	Y	Y	C	Y	6/7
Farmer et al^61^	Y	Y	Y	Y	Y	N	Y	6/7
Waller et al^18^	Y	Y	Y	Y	Y	N	Y	6/7
Bootwong and Intarut^62^	Y	Y	C	Y	Y	Y	Y	6/7
Singleton et al^63^	Y	Y	Y	Y	Y	Y	Y	7/7
Franco et al^64^	Y	Y	Y	Y	N	N	N	4/7
Zamanillo-Campos et al^65^	Y	Y	C	Y	Y	Y	Y	6/7
Kellner and Faas^67^	Y	Y	C	Y	N	C	N	3/7
Kellner et al^66^	Y	Y	C	Y	N	C	N	3/7
Golbus et al^68^	Y	Y	C	Y	Y	N	Y	5/7
Acevedo et al^20^	Y	Y	C	Y	Y	Y	Y	6/7

	Non-Randomised Quantitative (n = 6)							MMAT Scoring
	S1	S2	Q1	Q2	Q3	Q4	Q5	
McCoy et al^43^	Y	Y	N	N	Y	C	Y	4/7
Nepper et al^50^	Y	Y	Y	Y	Y	Y	Y	7/7
Gore et al^51^	Y	Y	Y	C	C	C	Y	4/7
Ritchie et al^60^	Y	Y	Y	Y	Y	Y	Y	7/7
Saldivar et al^27^	Y	Y	Y	Y	N	C	C	4/7
Huang et al^69^	Y	Y	Y	Y	Y	Y	Y	7/7

	Mixed Methods (n = 1)							
Keating and McCurry^54^	Y	Y	C	Y	Y	C	C	4/7
Criteria met (%)	100	100	33	97	72	38	72	

Abbreviations: Y, Yes-criteria met; N, No-criteria not met; C, Cannot tell if criteria was met.

Screening questions (for all types) S1. Are there clear research questions? S2. Do collected data allow to address the research questions?

Quantitative randomized controlled trials: 2.1. Is randomization appropriately performed? 2.2. Are the groups comparable at baseline? 2.3. Are there complete outcome data? 2.4. Are outcome assessors blinded to the intervention provided? 2.5 Did the participants adhere to the assigned intervention?

Quantitative non-randomized: 3.1. Are the participants representative of the target population? 3.2. Are measurements appropriate regarding both the outcome and intervention (or exposure)? 3.3. Are there complete outcome data? 3.4. Are the confounders accounted for in the design and analysis? 3.5. During the study period, is the intervention administered (or exposure occurred) as intended?

Mixed methods: 5.1. Is there an adequate rationale for using a mixed methods design to address the research question? 5.2. Are the different components of the study effectively integrated to answer the research question? 5.3. Are the outputs of the integration of qualitative and quantitative components adequately interpreted? 5.4. Are divergences and inconsistencies between quantitative and qualitative results adequately addressed? 5.5. Do the different components of the study adhere to the quality criteria of each tradition of the methods involved?

The lowest quality criterion observed for the RCTs included in the review was for randomization. For randomization to be appropriate, random allocation and allocation concealment need to be performed. More than half the studies (61%) did not provide enough information to determine whether allocation concealment was completed. Another criterion that was not met by more than half of the included studies was the blinding of outcome assessors, with 33% of studies not stating whether blinding occurred, and 28% having no blinding. Almost all studies had intervention and control groups that were comparable at baseline (97%). Most studies also met the criteria of having complete outcome data (participation retention rate of 80%), and participation adherence to assigned intervention (72%).

For the non-randomized studies, half of the studies did not give enough information to determine whether the analysis accounted for possible confounders.^27,43,51^ Most studies had samples that were representative of the population (83%) and had interventions that were administered as intended (83%).

## Publication Bias

**Figure 2.**
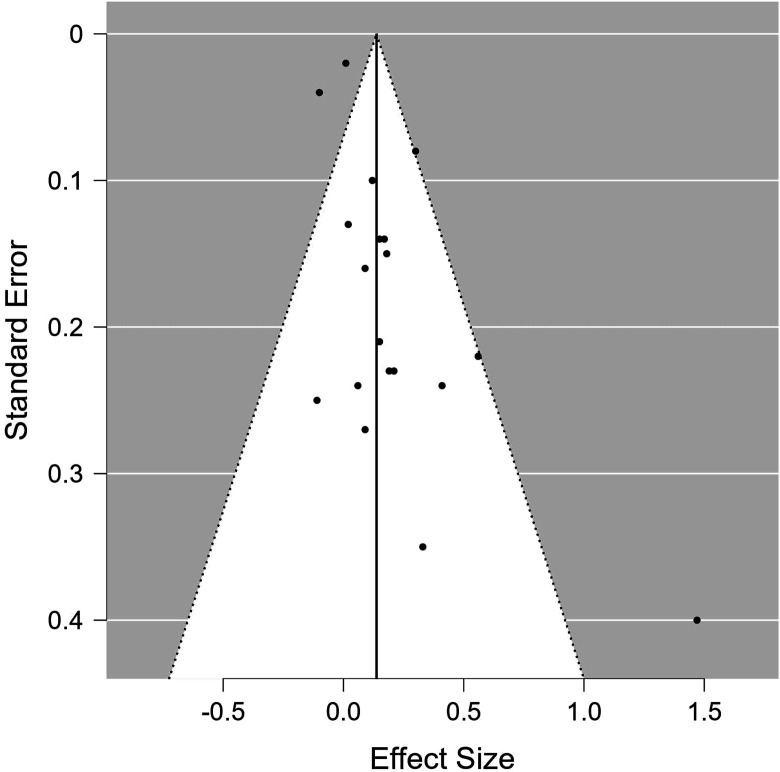
Funnel plot for publication bias of studies measuring physical activity outcomes.

**Figure 3.**
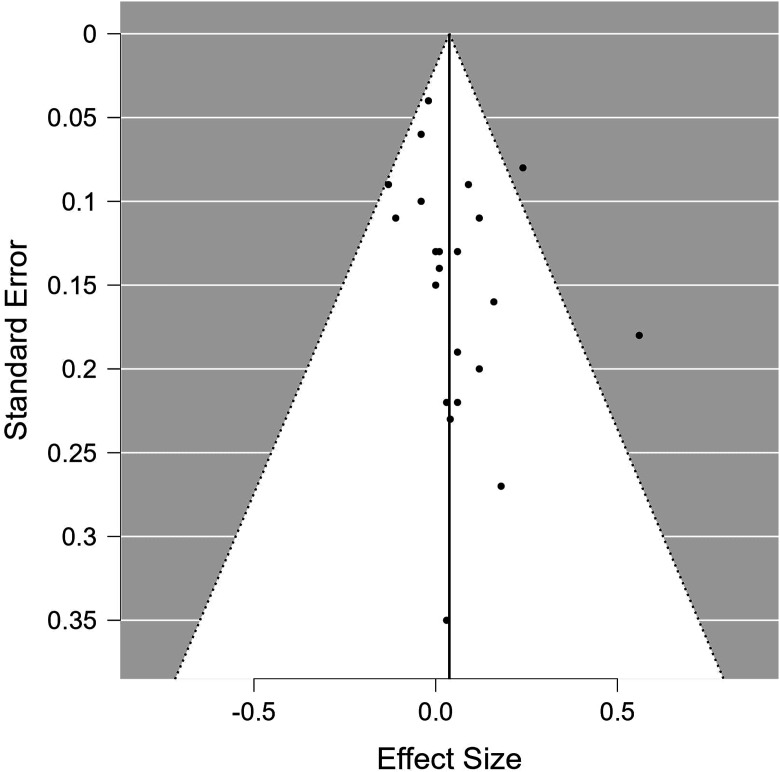
Funnel plot for publication bias of studies measuring weight-related outcomes.

Funnel plot analysis suggested no publication bias for studies measuring weight-related outcomes (Rank correlation test: *P* = .12, Egger’s test *P* = .19). Similarly, the rank correlation test suggested no publication bias for studies measuring physical activity (*P* = .53). However, potential bias was suggested by the Egger’s test (*P* = .004). (Figures 2 and 3).

## Meta-Analysis

**Figure 4.**
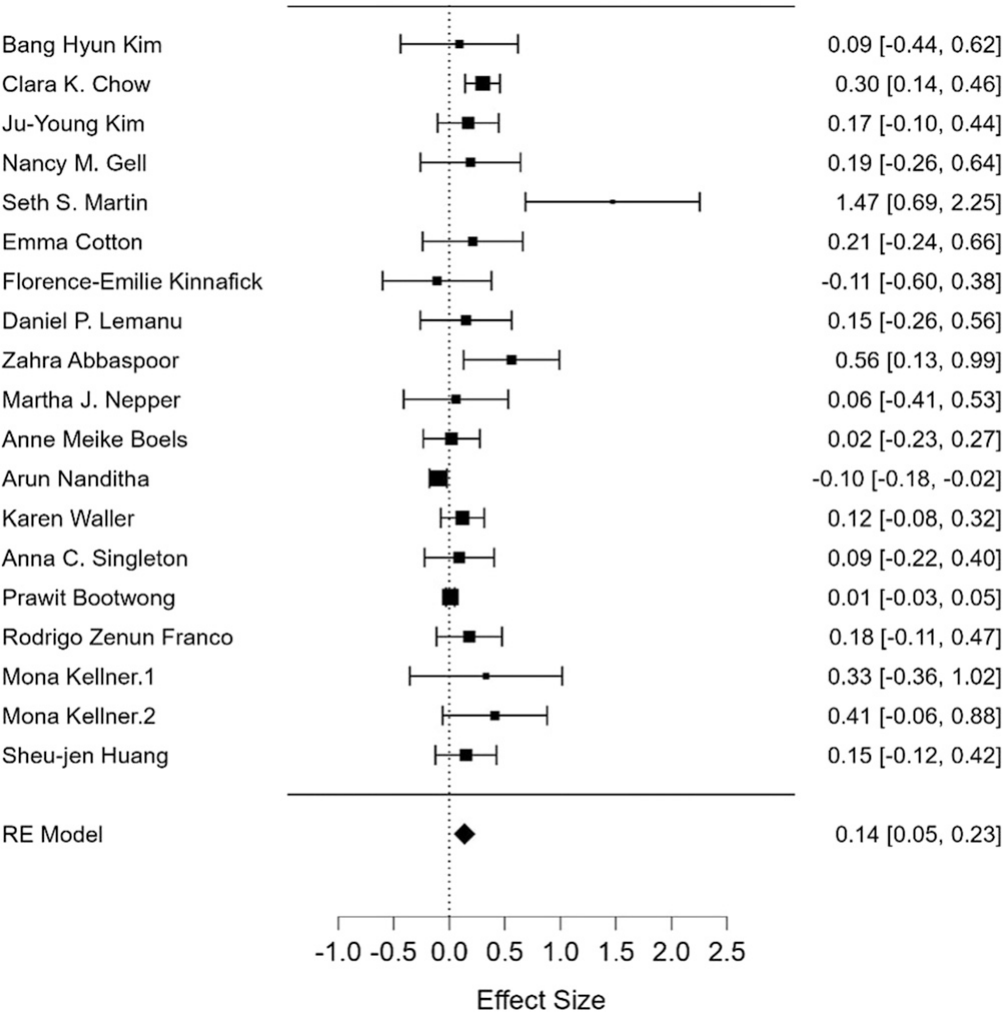
Forest plot showing meta-analysis of text messaging based mobile health interventions on physical activity.

**Figure 5.**
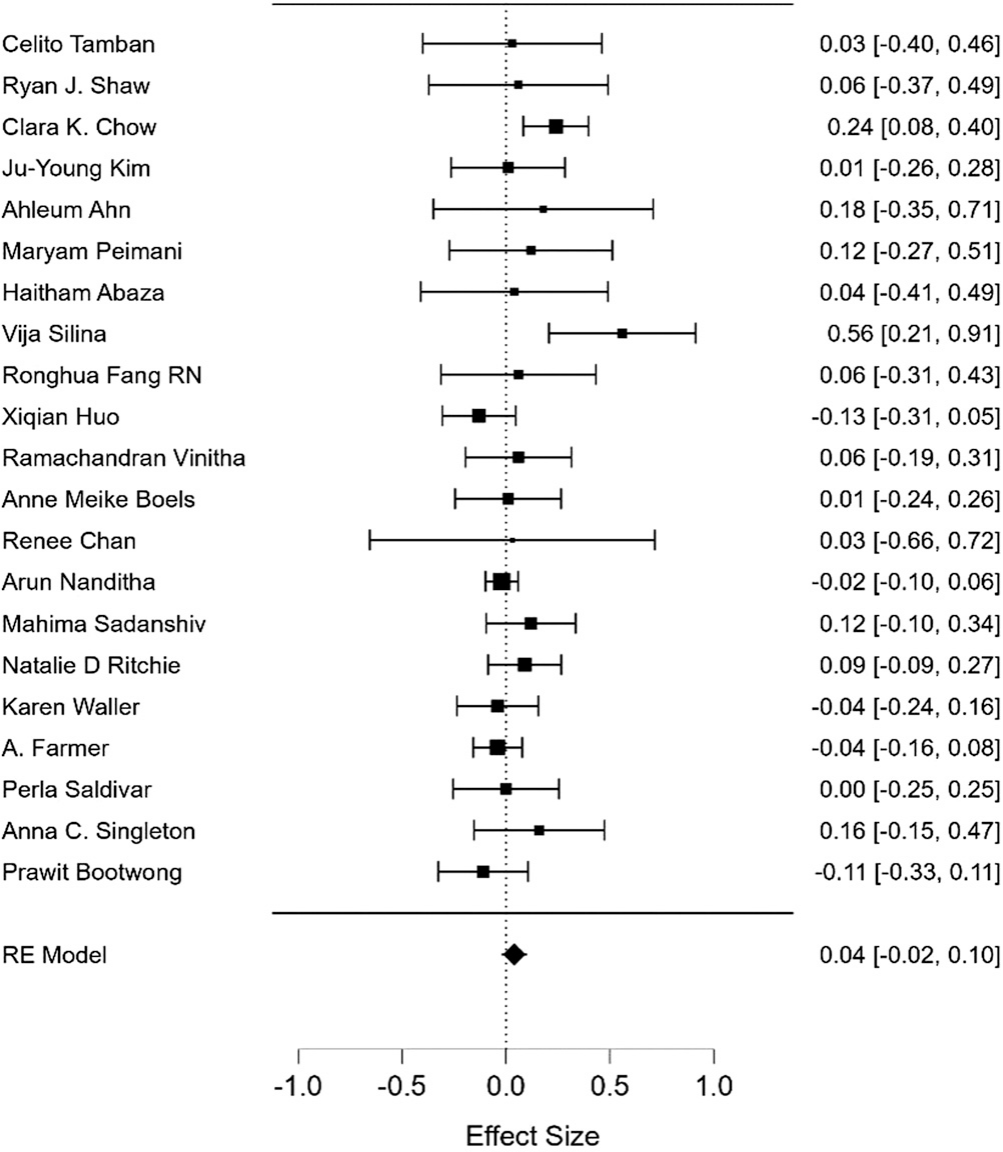
Forest plot showing meta-analysis of text messaging based mobile health interventions on weight-related outcomes.

A summary of the meta-analysis findings can be seen in Figures 4 and 5. A total of 19 studies were included for the physical activity meta-analysis and 22 studies for the weight-related outcome meta-analysis. Ten studies were excluded from meta-analysis due to insufficient data being available.

The pooled meta-analysis for physical activity-related outcomes was .14 (95% CI: .05 to .23, *P* = .003) and for weight-related outcomes .04 (95% CI: −.02 to .10, *P* = .21). Heterogeneity was high for physical activity (I^2^ = 65%), and moderate for weight-related outcomes (I^2 =^ 29%). This demonstrates that mHealth studies using unidirectional messaging had small effects on physical activity and no effects on weight-related outcomes.

For subgroup meta-analysis, statistically significant pooled effect sizes were observed for physical activity for studies using non-tailored messages [d+: .13 (−.02 to .22), *P* = .02], for studies with physical activity as a primary outcome [d+: .21 (.10 to .37), *P* < .001], non-theory-based messages [d+: .17 (.03 to .31) *P* = .01], and self-reported physical activity [d+: .14 (.05 to .23) *P* = .002]. However, the subgroup analysis yielded no statistically significant differences between these effect moderators (Appendix B).

The overall quality of evidence is presented in Table 3. The quality of evidence for studies included in the meta-analysis was scored low, due to lack of allocation concealment and blinding in the RCTs (as presented in MMAT appraisal). Additionally, most studies had small sample sizes which may lead to imprecision of intervention effects.

**Table 3.** Quality of Evidence.

Outcome	Number of Participants (Number of studies)^a^	Study Type Number	Standardized Mean Differences (95% CI)	Quality of Evidence (GRADE^b^)
Physical activity-related outcomes	8512 (19)	RCT: 17	.14 (.05 to .23)	Low^c,d^
		Non-RCT:2		
Weight-related outcomes	8326 (22)	RCT: 20	.04 (.02 to .10)	Low^c,d^
		Non-RCT:2		

^a^Studies used in meta-analysis.

^b^GRADE: Grading of Recommendations Assessment, Development, and Evaluation.

^c^Downgraded due to risk of biases (selection, detection, performance bias and blinding).

^d^Downgraded due to imprecision (small sample sizes).

## Sensitivity Analysis

Sensitivity analysis was conducted by removing effect outlier studies that were identified through funnel plot analysis. Pooled effects decreased slightly for both physical activity [d + .11 (.04 to .18), *P* = .007], and weight [d + −.01 (−.05 to .04), *P* = .80]. However, heterogeneity substantially decreased for both outcomes (physical activity I^2^ = 20%; Weight I^2^ = 0%).

A second sensitivity analysis was conducted by excluding the non-RCT studies, which are at higher risk of bias. When non-RCT studies were removed, effect sizes remained similar for both outcomes, while heterogeneity slightly increased [physical activity .14 (.04 to .25), *P* = .005, I^2^ = 70%; Weight .04 (−.03 to .10), *P* = .27, I^2^ = 35%].

## Discussion

The primary aim of this systematic review and meta-analysis was to determine the effect of unidirectional text message/instant message-based mHealth interventions on physical activity and weight-related outcomes.

The meta-analysis showed that text message-based mHealth interventions had very small pooled effects on physical activity outcomes [d+: .14 (95% CI: .05 to .23, *P* = .003)], and no effect on weight-related outcomes [d+: .04 (95% CI: −.02 to .10, *P* = .21)]. These findings are similar to other existing meta-analysis results. Jung and Cho^9^ conducted a meta-analysis on mHealth interventions promoting physical activity and weight loss amongst workers, and observed a pooled effect size for physical activity of .22 (95% CI .03 to .41; *P* < .001) and .02 (95% CI –.07 to .10; *P* = .48) for weight loss. Additionally, a meta-analysis of internet-based mHealth interventions observed a small pooled effect size on physical activity (d + .24, CI: .09 to .38).^21^

In contrast, a meta-analysis by Head et al observed effect sizes for physical activity of .51 and .25 for weight loss.^14^ However, the above compared meta-analyses were multimodal mHealth interventions that used other intervention components in addition to text messaging, such as face to face counseling, and thus are not completely comparable to the current review.

Therefore, it is evident that existing systematic reviews and meta-analyses have mixed findings, which mostly consist of results with small effects on physical activity and weight changes. It is also evident that text message-based studies are often heterogenous and vary in several intervention characteristics, thus limiting the quality of study comparisons.

The differences in intervention characteristics are important, as they may influence meta-analysis results. Other potential differences in intervention characteristics include message direction and the type of intervention modalities used. Our review included unidirectional, text message-only interventions, and found limited effects on physical activity and weight loss outcomes. This suggests that despite unidirectional messaging potentially being more straightforward and cost-effective for participants, it may not be as effective as bidirectional messaging. Limitations in unidirectional messaging include its lack of interactivity, which may make participation engagement more challenging, especially when the intervention is performed remotely.^12,17^

Nonetheless, a study by Sahin et al showed that unidirectional messaging had greater effects than bidirectional messaging interventions in participants with diabetes [unidirectional: d+ = .79 (.13-1.46); bidirectional: d+ = .27 (.10-.63). *P* = .022].^70^ Furthermore, the review observed greater effects in multimodal interventions compared to text message-only interventions [Multimodal: d+ = .79 (CI: .19-1.39) *P* = 0.013; text message only: d+ = .18 (CI: −.02 to .38)].^70^

In contrast, Head et al showed no differences in intervention effects of unidirectional and bidirectional messaging, and text-only vs text plus other interventions.^14^

Other potentially important effect moderators included tailoring messages, theory-based messages and primary vs secondary outcomes. For subgroup analysis, physical activity measured as a primary outcome had a statistically significant pooled effect size [d+: .21 (.10 to .37), *P* < .001]. This may be due to intervention messages generally being more targeted to primary outcomes compared to secondary outcomes. Self-reported physical activity also had a statistically significant pooled effect [d+: .14 (.05 to .23) *P* = .002]. This may be explained by the possibility of participants overreporting their physical activity post intervention.^71^ However, there were no differences between the pooled effects of primary vs secondary outcomes and self-reported vs objective outcomes.

Surprisingly, non-tailored and non-theory-based messages had statistically significant subgroup effect sizes for physical activity. This may partly be due to a larger sample size of studies using non-tailored messages (72%) being included in the review. Additionally, the type of theory used in included studies may have influenced the significance of the subgroup effect. In a review by Head et al that showed a statistically significant difference in the effects of theory-based messaging, most studies used the transtheoretical model and social cognitive theory.^14^ While only 6 of the 20 studies in our review used these aforementioned theories. Furthermore, there were no statistically significant differences between pooled estimates of tailored vs non-tailored, and theory vs non-theory-based interventions.

Previous reviews have demonstrated the benefits of using tailoring and theory-based messages. Head et al observed greater effect sizes for tailored and targeted messages when compared to non-tailored messages (d+ = .42 vs d+ = .27), and for theory-based interventions compared to non-theory-based interventions (d+ = .37 vs d+ = .27).^14^ However, not all studies have demonstrated this relationship. A study by Peimani et al failed to show that text messages tailored to individuals’ health barriers were more effective than general, non-tailored messages.^41^ Thus, knowledge gaps are still present as to whether certain intervention characteristics such as message tailoring have significant effects on study results.

Furthermore, this systematic review highlighted several potential gaps in mHealth literature. Only 12 of the 43 studies included in the review were conducted in LMICs. Existing systematic reviews have also reported a disparity between mHealth studies conducted in LMICs vs HICs.^8,9^ In a review on the effect of mHealth interventions on physical activity and weight loss, all 8 studies were conducted in HICs.^9^ While another systematic review exploring the effectiveness of mHealth interventions on physical activity, found that 106 out of 117 studies were conducted in HICs.^8^

Nevertheless, previous research has demonstrated the potential of mHealth interventions in LMICs, observing improvements in several NCD risk factors, including physical inactivity.^7^ Furthermore, according to the World Health Organization’s Global Status Report on Physical Activity, the use of mHealth programs in LMICs had increased from 10% in 2019, to 40% in 2021.^1^

Another potential gap in literature identified in this review, is the limited number of studies found using instant messaging for promotion of physical activity and weight loss. Only 4 studies included in this review used instant messaging as an mHealth medium. With the replacement of text messaging with instant messaging, as well as the rapid development of machine learning technologies, more studies using advanced communication mediums should be explored.^72^

## Strengths and Limitations

This systematic review and meta-analysis included instant message applications, which are more widely used in current times. Additionally, this review had more stringent inclusion criteria compared to existing systematic reviews on mHealth and lifestyle changes.^7,73^

However, despite the stricter inclusion criteria, study heterogeneity was moderate to high during primary meta-analysis. This may be due to several factors, such as population type, sample size variations, study duration, number of messages participants received, and the heterogeneity of text message content. However, when outlier studies were removed in sensitivity analysis, heterogeneity substantially decreased.

Another limitation in the review process was not including gray literature or unpublished studies in the search strategy, as this may influence meta-analysis results.^74^ Additionally, the literature search was only performed using 3 electronic databases, which may not have included all articles that met the inclusion criteria. Furthermore, using only articles published in English may have excluded important studies from the review, particular those conducted in LMICs.^75^

Lastly, the overall quality of the studies included in this review were scored low, due to many studies having small sample sizes, being unblinded, lacking allocation concealment and not accounting for potential confounding. These factors are important as they may result in a high risk of selection, performance, and detection bias and may distort the interpretation of results.^76,77^ The results of this review and meta-analysis should thus be understood with these limitations in mind, while still acknowledging its utility for guidance of future research.

## Conclusion

Based on the studies identified in this review, the use of unidirectional, message-only based mHealth interventions have small, albeit statistically significant effects on physical activity, and no effect on weight loss. These small effects may be due to unidirectional messages alone not being sufficient to elicit sustained behavior change. Studies using bidirectional messaging in addition to other interventions may have a greater effect on physical activity and weight-related outcomes. Additionally, although findings are equivocal, the use of theory and tailoring may also potentially improve effectiveness of such interventions. Lastly, this systematic review and meta-analysis have highlighted important gaps in mHealth literature. Few studies were identified that used instant messaging and that were conducted in LMICs. Thus, more research is required to address these limitations, and to ultimately determine whether mobile message-based interventions are an effective alternative to in-person interventions promoting physical activity and weight changes.

**CME/CE Article Quiz**

American College of Lifestyle Medicine (ACLM) members can earn FREE CME/CE credit by reading this approved CME/CE article and successfully completing the online CME/CE activity. Non-members can earn CME/CE for $40 per article. Visit lifestylemedicine.org to join the ACLM.

**Instructions.**

1. AJLM CME/CE Articles and Quizzes are offered online only through the American College of Lifestyle Medicine and are accessible at lifestylemedicine.org/store. ACLM Members can enroll in the activity, complete the quiz, and earn this CME/CE for free. Non-members will be charged $40 per article.
2. A Passing score of 80% or higher is required in order to be awarded the CME/CE credit.

## Supplemental Material

Supplemental Material - The Effect of Text Message Based mHealth Interventions on Physical Activity and Weight Loss: A Systematic Review and Meta-Analysis Supplemental Material for The Effect of Text Message-Based mHealth Interventions on Physical Activity and Weight Loss: A Systematic Review and Meta-Analysis by Aminah Emeran, Estelle Lambert, Robyn Burrows, Josh Loyson, Muhammed Rizaan Behardien, and Lauren Wiemers in American Journal of Lifestyle Medicine.
