# Supplementary material for: The Effect of Text Message-Based mHealth Interventions on Physical Activity and Weight Loss: A Systematic Review and Meta-Analysis
Source: Am J Lifestyle Med. 2024 Aug 15;20(6):952–74. doi: 10.1177/15598276241268324 (PMC11562155; doi:10.1177/15598276241268324)
Supplement: Supplemental Material - The Effect of Text Message Based mHealth Interventions on Physical Activity and Weight Loss: A Systematic Review and Meta-Analysis [file sj-pdf-1-ajl-10.1177_15598276241268324.pdf]

Supplementary Material:

Appendix A: Table showing Boolean search terms for each outcome.

**Outcome 1**	**Physical activity**
Search 1	((((((("physical activity") OR (exercise)) OR ("exercise training")) OR ("physical exercise")) OR ("physical training")) OR (steps)) OR ("sedentary lifestyle")) OR ("sedentary time")) OR ("physical inactivity")
Search 2	(((((((((mHealth) OR (m-health)) OR ("mobile health")) OR (eHealth)) OR (SMS)) OR ("text messag*")) OR ("instant messag*")) OR (WhatsApp)) OR (WeChat)) OR (messenger)
Search 3	(((intervention) OR (programme)) OR (program)) OR (initiative)
Search 4	(((#1) AND (#2) AND (#3) NOT (systematic review) NOT (meta-analysis) NOT (reviews) NOT (protocol)) Filters: English, Adult: 19+ years, from 2010 - 2022
**Outcome 2**	**Weight loss**
Search 1	(((("weight loss") OR (BMI)) OR ("body mass index")) OR ("weight reduction")) OR ("body weight")
Search 2	(((((((((mHealth) OR (m-health)) OR ("mobile health")) OR (eHealth)) OR (SMS)) OR ("text messag*")) OR ("instant messag*")) OR (WhatsApp)) OR (WeChat)) OR (messenger)
Search 3	(((intervention) OR (programme)) OR (program)) OR (initiative)
Search 4	(((#1) AND (#2) AND (#3) NOT (systematic review) NOT (meta-analysis) NOT (reviews) NOT (protocol)) Filters: English, Adult: 19+ years, from 2010 - 2023

Table showing number of article results for search 4, for each outcome and database

**Outcome**	**PubMed**	**Scopus**	**Web of Science**
Physical activity	2382	1632	5315
Weight loss	1422	429	1326
Total	3804	2061	6641

APPENDIX B: Subgroup Analysis of Effect Moderators

Subgroup	k	d+ (95% CI)	I^2	P value	Test for subgroup differences
**Physical activity**					
Tailoring	8	0.14 (-0.02 to 0.30)	74	0.08	0.92
Non-tailoring	11	0.13 (-0.02 to 0.22)	31	0.02*	
Theory based	8	0.15 (-0.05 to 0.35)	74	0.14	0.87
No theory	10	0.17 (0.03 to 0.31)	66	0.01*	
Self-reported	14	0.14 (0.05 to 0.23)	41	0.002*	0.29
Objective	5	0.31 (-0.16 to 0.79)	83	0.19	
Primary	11	0.21 (0.10 to 0.37)	0	<0.001*	0.28
Secondary	6	0.09 (-0.05 to 0.23)	70	0.20	
**Weight**					
Tailoring	7	0.05 (-0.05 to 0.15)	39	0.31	0.77
Non-tailoring	15	0.03 (-0.05 to 0.10)	22	0.45	
Theory based	9	-0.01 (-0.06 to 0.05)	0.005	0.75	0.16
No theory	13	0.07 (-0.01 to 0.15)	14	0.09	
Self-reported	3	0.02 (-0.21 to 0.25)	0	0.86	0.82
Objective	19	0.04 (-0.02 to 0.10)	35	0.21	
Primary	5	0.17 (-0.05 to 0.38)	50	0.13	0.11
Secondary	14	0.03 (-0.04 to 0.09)	32	0.44	

K: number of articles included in meta-analysis, d+: pooled effect size, * $p<0.05$
